# Safe Mobility in Hospitalised Older Adults: A Concept Analysis

**DOI:** 10.1111/jocn.70046

**Published:** 2025-07-27

**Authors:** Esther Mourão Nicoli, Frances Valéria Costa e Silva, Rosane Barreto Cardoso, Tallita Mello Delphino, Luciana Guimarães Assad, Samara Gonçalves de Oliveira

**Affiliations:** ^1^ Rio de Janeiro State University Rio de Janeiro Brazil; ^2^ Federal University of Rio de Janeiro Rio de Janeiro Brazil

**Keywords:** aged, hospitals, mobility, safety

## Abstract

**Aim:**

To conduct a concept analysis of ‘safe mobility’, with specific application in hospitalised older adults, identifying its defining attributes, antecedents and consequences.

**Background:**

The promotion of safe mobility is essential for maintaining the functionality of hospitalised older adults. However, this idea is not yet clearly defined in the scientific literature, requiring a conceptual analysis for better understanding and applicability in nursing practice.

**Design:**

Concept analysis.

**Methods:**

The concept analysis methodology of Walker and Avant was employed, consisting of eight steps. Sources from the scientific literature (BDENF/VHL, Scopus, CINAHL/EBSCO, Embase, Web of Science, PEDro, MEDLINE/PubMed and CAPES Thesis and Dissertation Catalogue, as outlined in a scoping review previously published by the authors) and terminologies from dictionaries and nursing practice, such as SNOMED CT, ICNP, NANDA, NIC and NOC, were analysed.

**Results:**

The concept of ‘safe mobility’ does not have a consolidated definition but was identified through three defining attributes: active movement, prevention of fall‐related harm and prevention of immobility‐related harm. The antecedents include the older adults' conditions, adaptation of the hospital environment, training of the multidisciplinary team, patient behaviour and family involvement. The consequences involve the maintenance of functionality, improvement of quality of life, reduction of hospital length of stay and costs, as well as a decrease in rates of readmission, referrals to long‐term care institutions and mortality.

**Conclusion:**

The concept analysis revealed that safe mobility involves promoting active movement and preventing harm related to both immobility and falls.

**Implications for the Profession and Patient Care:**

Strategies based on this concept can improve the quality of life of older adults, reduce complications and optimise hospital costs.

**No Patient or Public Contribution:**

This concept analysis examines existing literature and does not require patient‐related data collection. The methodological approach does not necessitate collaboration with the public.

**Summary**

- The study addressed the lack of a clear and consolidated definition of the concept of "safe mobility" in the context of hospitalized older adults, which limits its application in nursing practice.
- The main findings identified three defining attributes of safe mobility: active movement, prevention of fall‐related harm, and prevention of immobility‐related harm. The study also mapped antecedents (such as patient conditions, environment adaptation, team training, patient behavior, and family involvement) and consequences (like maintained functionality, improved quality of life, reduced hospital stay, lower healthcare costs, fewer readmissions, decreased institutionalization, and reduced mortality).
- The research will impact clinical practice in hospital settings, particularly in the care of hospitalized older adults. It provides healthcare professionals, especially nurses, with a clearer conceptual framework to design, implement, and evaluate interventions that promote safe mobility, ultimately benefiting patients, healthcare teams, and health systems.

## 1 Introduction

The natural process of ageing leads to changes that can impair mobility, such as loss of skeletal muscle mass and strength (Olson et al. 2022). After the age of 50, about 0.5%–1% of muscle mass can be lost annually, even considering lifestyle and genetic factors (Di Girolamo et al. 2021). In the hospital setting, this loss can be exacerbated by clinical conditions, therapeutic procedures or fall prevention measures that further restrict mobility, as well as by a hospital culture that considers bed rest a natural part of hospitalisation (Johnston and Magnan 2019).

Older patients admitted for treatment of acute illness spend an average of 17 h lying down, 5.1 h sitting, and take an average of only 728 steps per day during hospitalisation—while the recommended amount is at least 4600 steps per day,—even though they may have the potential to be more physically active (Piper et al. 2024). Prolonged immobility can exacerbate motor unit denervation and neuromuscular junction degeneration, contributing to functional and cognitive decline, increased risk of falls and fractures, geriatric syndromes, rehospitalisation and even death. This makes low in‐hospital activity a significant risk factor for the development of hospital‐associated disabilities (HADs), which often leads to institutionalisation in a nursing home after discharge (Hendrich 2021). In this context, it is understandable why mobility is one of the Geriatric 5 M's—a framework for assessing and acting on 5 critical issues in the care of older adults: Mind, Mobility, Medications, Multi‐complexity, Matters most (Molnar 2017).

Given the importance of interventions that promote mobility in hospitalised older adults, and their increased risk of falls—without disregarding that falls are a common, serious and growing public health issue, and one of the leading causes of morbidity and mortality in older adults—the idea of ‘safe mobility’ emerges (Kumble et al. 2024). This approach seeks not only to encourage mobility during hospitalisation, but also to ensure that such mobility occurs safely, considering the potential harm caused by falls (Kumble et al. 2024). However, the concept ‘safe mobility’ lacks clarity in nursing literature: a scoping review mapping existing studies on the topic found that none of the reviewed articles provided a clear definition of the term ‘safe mobility’ (Nicoli et al. 2024). The term ‘mobility’, itself, has been frequently used in various ways: (1) to describe the physical activities necessary to maintain the functional ability (e. g. ambulation, range‐of‐motion and strengthening exercises); (2) to describe the physical functional ability (what the patient is actually able to do); (3) to quantify levels of mobility (e.g., low level of mobility has been described as inactivity) (Smart et al. 2018).

The literature reveals some confusion regarding the use of terms such as ‘safe mobility’, ‘safe mobility behavior’, ‘safe gait’, ‘safe patient handling equipment’, ‘early mobility’, ‘functional mobility’ and ‘progressive mobility’ (Nicoli et al. 2024), which raises important questions: At what point do these terms become interchangeable? Is safe mobility for hospitalised older adults also safe for the healthcare team? Considering that the promotion of safe mobility is a multidisciplinary intervention, what are the specific roles and responsibilities of the nursing staff (nurses and nursing technicians)? What organisational factors are essential to support its implementation? These questions highlight the need for a concept analysis, a method that examines the structure and application of a term, facilitating its understanding, use in professional practice and guide future research (Walker and Avant 2019).

In a context of population aging and increasing hospital complexity—in Western countries, older patients account for approximately 40% of admissions in medical and surgical wards—clarifying the concept ‘safe mobility’ provides a necessary theoretical scaffold for evidence‐based delineation of its scope, guides clinical decision‐making, proposes a nursing intervention, and supports the transition from a model focused solely on fall prevention to an integrated approach centred on rehabilitation and maintaining functionality in hospitalised older adults (Olson et al. 2022). This relevance lies in the fact that maintaining or improving the functionality of older adults not only promotes better clinical outcomes but also significantly reduces hospital costs, which is essential for the sustainability of healthcare systems (Okamoto et al. 2023). Given this context, this study aimed to conduct a concept analysis of ‘safe mobility’, with specific application in the hospitalised older adults, identifying its defining attributes, antecedents and consequences.

## 2 Methods

### 2.1 Concept Analysis Method

A concept analysis was conducted using Walker and Avant's method, which aims to clarify the meaning of a concept by breaking it down into its defining attributes, uses and implications (Walker and Avant 2019). The method follows eight systematic steps: (1) Select a concept—Choose a concept of interest that requires clarification; (2) determine the aims or purpose of the analysis—Define why the analysis is being conducted and what it hopes to achieve; (3) identify all uses of the concept—explore how the concept is used in various contexts and disciplines; (4) determine the defining attributes—identify the essential characteristics that appear repeatedly in the literature; (5) construct a model case—create an example that includes all the defining attributes of the concept; (6) construct borderline, related, contrary, invented or illegitimate cases—Develop other types of cases to clarify what the concept is and is not; (7) identify antecedents and consequences—Examine what conditions must occur before the concept (antecedents) and what results from it (consequences); (8) define empirical referents—determine how the concept can be observed or measured in practice (Walker and Avant 2019). This method helps build conceptual clarity and supports theoretical and practical applications in research and clinical settings (Walker and Avant 2019). Its approach was chosen due to its suitability for systematically clarifying a concept, exploring it as a potential nursing intervention for hospitalised older adults.

### 2.2 Data Source

A scoping review (SR) published by the authors was used to map the application of ‘safe mobility’, in hospitalised older adults (Nicoli et al. 2024). Additionally, dictionary terminologies and nursing practice classifications were reviewed, including: **SNOMED CT** (Systematised Nomenclature of Medicine—Clinical Terms, a comprehensive, multilingual clinical terminology used globally to encode health information across all medical disciplines), **ICNP** (International Classification for Nursing Practice, developed by the International Council of Nurses, that provides a unified nursing language and supports the description and comparison of nursing practices worldwide), **NANDA‐I** (NANDA‐International, a taxonomy focuses on standardised nursing diagnoses), **NIC** (Nursing Interventions Classification, that offer standardised terms for nursing interventions) and **NOC** (Nursing Outcomes Classification, that offer standardised terms for patient outcomes). These sources were selected for this concept analysis as they represent internationally recognised standardised nursing and healthcare classifications that facilitate accurate clinical documentation, promote effective interdisciplinary communication, and support the practice.

The cited scoping review—‘Nursing care for hospitalized older adults – fall accidents versus safe mobility: a scoping review’—was guided by JBI guidelines, an international research organisation that guides systematic reviews (Nicoli et al. 2024). Valuing the writing quality and smoothness of this study, the Preferred Reporting Items for Systematic reviews and Meta‐Analyses extension for Scoping Reviews (PRISMA‐ScR) checklist guidelines were followed (Supporting Information File 1) (Nicoli et al. 2024). The eligibility criteria are linked to the PCC acronym structure—P (population/participant), C (concept) and C (context). For population/participant ‘P’, older adults—individuals aged 60 or older, of both sexes were included. For concept ‘C’, studies that define, report or provide information on safe mobility—relevant approaches that help or encourage older adults to move safely daily—and the factors associated with promoting this were included. For context ‘C’, studies that involve the care of hospitalised older adults, in multiple circumstances (clinical, surgical, among others), covering public or private hospitals, small, medium or large, teaching, general, specialised, urban or rural, were included (Nicoli et al. 2024).

The scoping review considered primary research studies, reviews and case reports, with a quantitative or qualitative design. Furthermore, reports, institutional texts with relevance in geriatrics/gerontology, books and guidelines published in indexed sources consulted or in grey literature were included. Articles published only as abstracts, letters to editor and comments were excluded. No time and language cut‐off were established.

**FIGURE 1:**
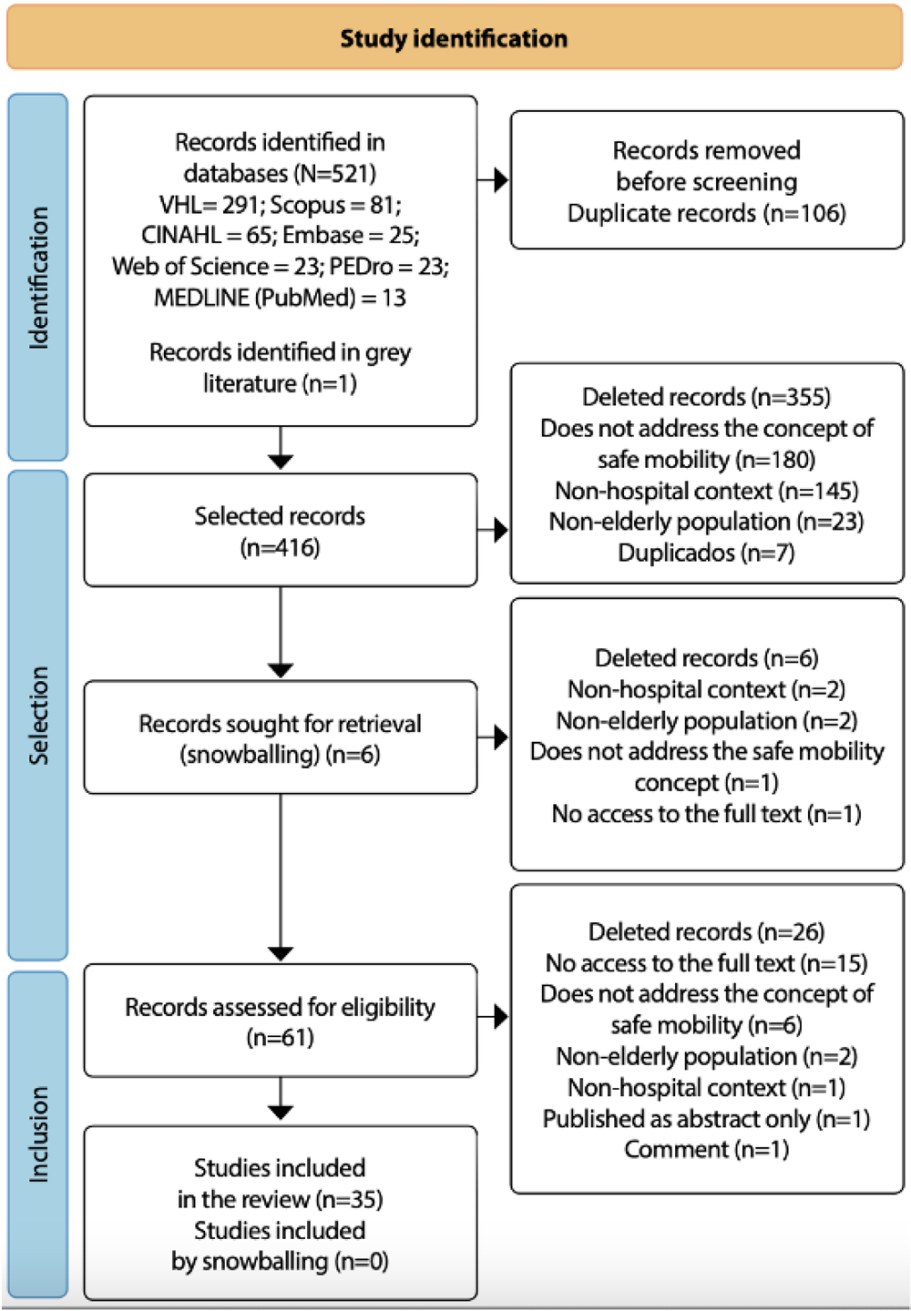
PRISMA SR flowchart for identification, selection and inclusion of studies containing constituent elements of the safe mobility concept in hospitalised older adults, Rio de Janeiro, Rio de Janeiro, Brazil, 2023. *Source:* Nicoli et al. (2024). [Colour figure can be viewed at wileyonlinelibrary.com]

The search was conducted in the following sources: BDENF/VHL (Virtual Health Library), Scopus, CINAHL/EBSCO, Embase, Web of Science Core Collection, PEDro, MEDLINE/PubMed and the CAPES Thesis and Dissertation Catalogue; no temporal or language restrictions were applied (Nicoli et al. 2024). The search sources were selected in collaboration with a librarian to ensure the research would be as comprehensive as possible, not limited to the field of nursing. Studies were selected by two independent reviewers, and in case of disagreement, a third researcher was consulted (Nicoli et al. 2024). The scoping review included 35 works, as shown in Figure 1, available in the previously published research (Nicoli et al. 2024).

An additional search was conducted on January 6, 2025, using the same strategy and databases as the initial search, in order to identify any possible new publications since the completion of the SR in 2023—complete search strategy for CINAHL, which was adapted to the other databases: (MM “Aged”) OR (MM “Aged, 80 and Over”) OR aged OR “aged patient” OR “aged people” OR “aged person” OR “middle aged” OR elderly OR “elderly patient” OR “elderly people” OR “elderly person” OR “elderly subject” OR “senior citizen” OR senium) AND (“fall risk” OR “Accidental Falls” OR “Accidental Fall” OR “Fall and Slip” OR Fall‐ ing) AND “safe mobility” OR mobility AND (MM “Hospitalization”) OR (Hospitalisation OR Hospitalisations OR Hospitalizations). No articles meeting the study's inclusion criteria were found.

The data extracted from the 35 studies included on the scoping review, on dictionary terminologies and nursing practice classifications were organised to identify conceptual and empirical patterns regarding the promotion of safe mobility in hospitalised older adults. The findings were systematically categorised into defining attributes, antecedents and consequences, in accordance with the principles of concept analysis methodology.

### 2.3 Rigour

The reliability of findings was ensured through peer review and data triangulation. Study selection followed predefined criteria, and analyses were validated by experienced researchers in gerontological nursing. Walker and Avant's (2019) methodology provided a structured and standardised framework, enhancing the precision of concept characterisation.

## 3 Results

The initial two steps of the concept analysis—(1) selecting a concept and (2) determining the purpose of the analysis—are outlined in the Introduction section. Consequently, the Results section will address steps three through eight.

### 3.1 Uses of the Concept

This stage of analysis requires evaluating and synthesising conceptual examples (Walker and Avant 2019). Dictionary, terminology and scientific literature definitions of the concept were described.

#### 3.1.1 Dictionary and Terminology Definitions

According to the Cambridge Dictionary, online version, ‘mobility’ refers to ‘the ability to move or walk around freely’ and ‘safe’ means ‘not dangerous or likely to cause harm/used to refer to things that do not involve any risk’ (Cambridge Dictionary 2025). In a search conducted in SNOMED CT, 2025 edition, international version, the concept ‘safe mobility’ (code 225986002) is defined as ‘gaining safe level of mobility prior to hospital discharge (procedure)’ (SNOMED CT, [s. d.] n.d.). The term ‘safe mobility’ was not explicitly identified in ICNP, nor in the latest versions of NANDA, NIC and NOC. ICNP includes the terms ‘promote physical mobility’ (10037379) and ‘safety’ (10032676).

In the 13th edition of NANDA‐I Nursing Diagnoses (2024–2026), mobility is classified under Domain 4 as the independent and intentional movement of the body or one or more extremities. Safety is addressed in Domain 11 and is related to being free from danger, physical injury or harm to the immune system; preservation against loss; and protection of security (Herdman et al. 2024).

The 7th edition of NIC, in Domain 1, Class C—immobility management, includes interventions aimed at controlling movement restrictions and their sequelae. Meanwhile, in Domain 4—safety, it encompasses care that supports protection against harm (Butcher et al. 2020). Additionally, in NIC, the intervention ‘Dementia Management’ (6460) mentions the activity ‘ambulate safely’ by proposing a modified environment for patients experiencing chronic confusion (Butcher et al. 2020). In the intervention ‘Dementia Management: Wandering’ (6466), the proposed activities include ‘providing a safe place for wandering’ and ‘modifying unsafe aspects’ (Butcher et al. 2020). The term mobility is also employed in the context of ‘joint mobility’, referring to the use of active and passive body movements to maintain or restore joint flexibility (Butcher et al. 2020).

**FIGURE 2:**
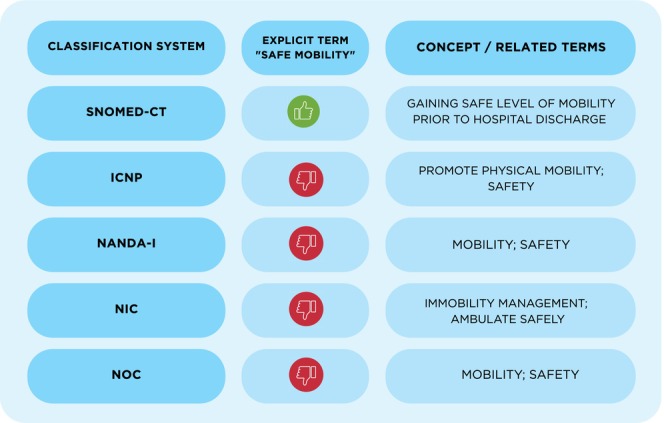
Presence or absence of the term ‘safe mobility’ on classification systems. 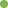: yes, there is an explicit mention of the term ‘safe mobility’; 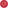: no, there is no explicit mention of the term ‘safe mobility’. *Source:* The authors, 2025. [Colour figure can be viewed at wileyonlinelibrary.com]

The 7th edition of NOC defines mobility (0208) as the ability to move purposefully within one's environment, independently, with or without an assistive device, and safety as behaviours or an individual's state that promotes protection against harm (Moorhead 2024). Figure 2 shows a brief table that summarises and contrasts the presence or absence of the term ‘safe mobility’ on classification systems.

#### 3.1.2 Use in the Scientific Literature

The use of the concept of ‘safe mobility’, with specific application in the hospitalised older adults, was mapped in the scientific literature through a scoping review (SR) published by the authors in 2024 (Nicoli et al. 2024). The review demonstrated either implicit (88.5%, *n* = 31) or explicit (11.5%, *n* = 4) use of the concept, based on multiprofessional approaches involving nursing, medicine, physical therapy, nutrition and occupational therapy (Nicoli et al. 2024). The SR found that the concept of safe mobility is not precise, but identified constitutive elements related to the patient, the institution and the nature of the interventions (Nicoli et al. 2024). The research also revealed the use of the term ‘safe mobility’ related to urban mobility, in the context of traffic (Nicoli et al. 2024).

Another study, a scoping review conducted by Australian researchers and also published in 2024, aimed to map the definition and concepts of safe mobility behaviour to extract implications for people with Parkinson's disease (Cheung et al. 2024). This study did not meet all the inclusion criteria of the present concept analysis—specifically, its context was community‐based rather than hospital‐based. However, it is noteworthy that the review defined safe mobility behaviour as any protective action and associated functional cognitive processes used to reduce the likelihood of a fall during mobility‐related activities (Cheung et al. 2024).

### 3.2 Defining Attributes

This stage is the core of the analysis, focusing on highlighting the set of attributes most frequently associated with the concept (Walker and Avant 2019). In summary, the aim is that, when mentioned, the defining attributes should immediately bring the concept to mind (Walker and Avant 2019). Table 1 presents the defining attributes of the concept of ‘safe mobility’, with specific application in the hospitalised older adults, based on the literature reviewed.

**TABLE 1:** Defining attributes of the concept of ‘safe mobility’, with specific application to hospitalised older adults, identified in the literature.

Defining attributes	Studies in which the defining attribute was identified
**1. Active movement:** Encouragement of strengthening, balance, stretching and aerobic exercises, walking, physical therapy sessions, activities of daily living (ADLs) and socialisation	Kneafsey et al. (2013); Mahoney (1998); Labella et al. (2011); Courtney et al. (2011); Laybourne et al. (2011); Said et al. (2012); Golder et al. (2012); Boltz et al. (2014); Lyons (2014); Nogueira Lira et al. (2015); Brown et al. (2016); Raymond et al. (2017); Wald et al. (2019); Aarden et al. (2019); Braun et al. (2019); Hartley et al. (2020); Mccullagh et al. (2020); Gazineo et al. (2021); Seeger et al. (2021); Sinha and Detsky (2012); Opasich et al. (2010); Nelson et al. (2004); Aizen et al. (2015); Zieschang et al. (2010); Resnick and Boltz (2019); Lim et al. (2020)
**2. Prevention of fall‐related harm:** Implementation of measures to minimise physical harm (serious injuries such as fractures, lacerations and concussions), as well as psychological and social consequences	Nogueira Lira et al. (2015); Mahoney (1998); Nelson et al. (2004); Sinha and Detsky (2012); Brown et al. (2007); Krause and Von Renteln‐Kruse (2007); Laybourne et al. (2011); Boltz et al. (2014); Lyons (2014); Aizen et al. (2015); Villafañe et al. (2015); Oliveira et al. (2016); Raymond et al. (2017); Wald et al. (2019); Resnick and Boltz (2019); Hendrich (2021); Seeger et al. (2021); Mudge et al. (2022); Zieschang et al. (2010); Golder et al. (2012); Kneafsey et al. (2013); Brown et al. (2016); Braun et al. (2019); Cerilo and Siegmund (2022)
**3. Prevention of immobility‐related harm:** Implementation of measures to prevent adverse effects on the muscular, skeletal, joint, integumentary, cardiovascular, respiratory, gastrointestinal, genitourinary, metabolic, cognitive and psychiatric systems, as well as postural control	Mahoney (1998); Nelson et al. (2004); Golder et al. (2012); Sinha and Detsky (2012); Kneafsey et al. (2013); Boltz et al. (2014); Lyons (2014); Nogueira Lira et al. (2015); Wald et al. (2019); Aarden et al. (2019); Resnick and Boltz (2019); Lim et al. (2020); Gazineo et al. (2021); Hendrich (2021); Seeger et al. (2021); Mudge et al. (2022)

*Source:* The authors, 2025.

Considering that the concept under analysis is ‘safe mobility’, the defining attributes presented are directly related to it. The specific context of hospitalised older adults determined the selection of these attributes, as it influences and modifies their expression. For example, immobility‐related harm has different impacts on older adults compared to younger adults or children; in a nonaged population, would the prevention of immobility‐related harm still be considered a defining attribute? Therefore, it is not possible to state that the findings are applicable to other populations or settings.

The first defining attribute, **active movement**, can be promoted through physiotherapy sessions and structured exercise programmes which may be proposed by the multidisciplinary team, particularly nursing—including strengthening, stretching, balance improvement or aerobic capacity enhancement—engagement in daily living activities or social interactions. Strengthening exercises aim to maintain muscle strength through frequent repetitions against gravitational force, focusing on dorsiflexors and plantar flexors of the ankle, knee extensors, hip extensors, as well as hip abductors and adductors (Zhong et al. 2024). Stretching exercises involve moving a joint through its full range of motion to prevent contractures, emphasising hip extension, knee extension, ankle dorsiflexion and shoulder flexion (Seppala and van der Velde 2023). Balance improvement exercises may include training on stabilometric platforms (Villafañe et al. 2015). Aerobic exercises begin with activities that mobilise cardiovascular capacity in a seated position and progress to bed‐to‐chair transfers, encouragement of walking to the bathroom, and, depending on tolerance, longer and more frequent walks (Seppala and van der Velde 2023).

The assessment of mobilisation tolerance considers signs and symptoms (fatigue, dyspnoea, requests for rest, a drop in systolic blood pressure by 10 mmHg, an increase in diastolic blood pressure by 15 mmHg, an increase in heart rate by 20 bpm during rest, failure of heart rate to return to baseline after 3 min of rest, and oxygen saturation dropping below 90%) that indicate activity should be discontinued, as the intensity level should remain light (Mahoney 1998). Mobility should be maintained with appropriate precautions, including supervision and involvement of a multidisciplinary team (Seppala and van der Velde 2023). Active movement can also be encouraged by promoting the continuation of routine tasks, as individuals would do at home, such as dressing, eating and going to the bathroom, as well as engaging in social and recreational activities, including communal meals and out‐of‐room activities such as physical activity‐based bingo (Resnick and Boltz 2019; Sinha and Detsky 2012).

However, as active movement is promoted, the risk of falling inevitably arises. Therefore, the second defining attribute identified is the **prevention of fall‐related harm**. Falls are considered undesirable events as they may cause potential physical harm (such as fractures—especially hip fracture—concussions, abrasions, hematomas, lacerations and prolonged immobility), psychological distress (fear of falling leading to insecurity in mobility) and social consequences (such as isolation, which is a known risk factor for dementia), ultimately delaying treatment progress, particularly in older adults (Couch 2019). Nevertheless, it is important to note that the defining attribute selected was ‘prevention of fall‐related harm’, rather than ‘fall prevention’ itself. This distinction is crucial because only 1.4% of falls result in fractures, indicating that most falls do not lead to serious physical harm. In contrast, restrictive measures, commonly used to prevent falls in hospital settings—such as raising all bed rails and instructing patients not to get out of bed—are always counterproductive for older adults, as they may lead to further physical and functional decline (Krause and Von Renteln‐Kruse 2007).

In this regard, the third defining attribute is related to the **prevention of immobility‐related harm**: damage to the muscular system (loss of strength and muscle mass, reduction in oxidative capacity), skeletal system (bone loss), joint system (reduced range of motion), integumentary system (pressure injuries), cardiovascular system (decreased workload capacity and postural hypotension), respiratory system (V/Q disturbances, atelectasis, reduced ventilatory capacity), gastrointestinal system (loss of appetite and constipation), genitourinary system (incomplete bladder emptying, increased calcium saturation), metabolic system (insulin resistance), cognitive function (perceptual changes, cognitive decline), psychiatric health (depression, anxiety) and postural control (increased body sway), which can predispose to geriatric syndromes.

Since promoting mobility inevitably involves some risk of falls and since the adverse physiological consequences of mobility restriction can also increase fall risk, the safe mobility approach aims to prevent severe injuries resulting from falls rather than to avoid all falls altogether (Cameron et al. 2024). In this sense, safe mobility promotion should not be reduced to fall prevention but should instead be understood as the promotion of conditions that enable active movement and reduce risks to the functional capacity of older adults (Resnick and Boltz 2019).

### 3.3 Cases

#### 3.3.1 Model Case

A model case is a clear and complete example of a concept that illustrates all of its defining attributes (Walker and Avant 2019). It serves as a paradigmatic instance, meaning that if this is not an example of the concept, then nothing is. The following case is fictional, designed by the authors to illustrate the concept ‘safe mobility’. The manifestation of the defining attributes was highlighted and identified in parentheses.

Mr. S.O., a 70‐year‐old healthy individual, was admitted to the ward from the emergency department of the same hospital. Upon admission, he was using diapers, had two peripheral venous accesses and was wearing flip‐flops. In the ward where he was transferred, the healthcare team was trained in gerontological care and remained vigilant and responsive to his needs. The patient was placed in a low, locked bed, with the upper side rails raised for safety, while the distal rails were left lowered to facilitate his movement. The bed had an adjustable height. His condition stabilised early, and the team encouraged early ambulation (active movement) as tolerated, instructing him to move slowly when getting out of bed. His medication prescriptions were reviewed, reducing polypharmacy, and one of the venous accesses was removed (prevention of fall‐related harm). His diet was adjusted to ensure adequate nutritional intake for his condition (prevention of fall‐related harm). The diaper was removed, and he was encouraged to perform basic activities of daily living, such as going to the bathroom independently (prevention of immobility‐related harm). Additionally, he was encouraged to participate in communal meals, as the hospital environment was equipped with signage, nonslip flooring, handrails and call alerts, among other safety supports (prevention of fall‐related harm). Following guidance from the healthcare team, the patient's family brought him a closed‐toe sandal with a firm sole and adjustable straps to facilitate safe walking. He was also provided with a properly fitted pyjama set that sufficiently covered his body (prevention of fall‐related harm). The family, encouraged by the healthcare team, actively participated in the process and motivated the patient to remain active (active movement).

Identify additional cases—contrary, borderline, related, invented and illegitimate—helps clarify which attributes truly define the concept by comparing examples that are similar to, overlap with or differ from the concept of interest (Walker and Avant 2019). These cases can come from real life, literature or be constructed to refine the understanding of the concept's defining characteristics (Walker and Avant 2019). Below, additional case models related to the concept under analysis will be presented.

#### 3.3.2 Contrary Case

A contrary case is a clear example of what the concept is not, helping to clarify its meaning by contrast (Walker and Avant 2019). It contains none of the defining attributes and highlights how the concept differs from similar or related ideas, making it easier to refine and confirm the essential characteristics of the concept being analysed (Walker and Avant 2019). Below is a fictional case designed by the authors:

Mr. S.O., a 70‐year‐old patient, was admitted to a ward with beds that did not allow height adjustment. He was instructed not to leave the bed under any circumstances due to the risk of falls and fractures, and all four bed rails were raised. The healthcare team prescribed the use of diapers, justifying the decision based on the patient's age and the staff's workload, which prevented them from assisting him to the bathroom. Concerned about the risk of falls, the family complied with the healthcare team's recommendations and reinforced the restriction, preventing the patient from engaging in any activity. The mobility restrictions led to disorientation and irritability, resulting in the use of mechanical restraints to keep Mr. S.O. in bed. His medication regimen and the number of treatment‐related devices increased significantly, as did complications, including multiple pressure ulcers and infections. After 2 months of hospitalisation, Mr. S.O. was discharged in a bedridden state, fully dependent on support for all activities of daily living.

#### 3.3.3 Borderline Case

Borderline cases are examples that include most of the defining attributes of a concept but differ in a key aspect, making them partially inconsistent with the concept being analysed (Walker and Avant 2019). These cases help clarify the boundaries of the concept by showing why certain examples do not fully meet its defining characteristics (Walker and Avant 2019). Below is a real case:

Mr. J. A. H., a 75‐year‐old man, was admitted to a surgical ward for postoperative care following a hip arthroplasty. He was alert and oriented, although slightly anxious. Upon arrival, the nursing staff assessed his mobility and encouraged progressive mobilisation starting on the first postoperative day (active movement). He was assisted in sitting in a chair and initiating ambulation with a walker (prevention of fall‐related harm). The environment included handrails and nonslip flooring, and the staff emphasised the use of appropriate footwear and the call bell (prevention of fall‐related harm). However, due to the patient's fear of falling—worsened by a previous fall at home—he was reluctant to get out of bed without a staff member present. As the ward was understaffed, his mobility was significantly reduced. Although there were no fall incidents and the team provided adequate instruction and environmental safety measures, his prolonged inactivity during certain periods of the day led to a decline in muscle strength and early signs of pressure injury on his sacral region (immobility‐related harm).

#### 3.3.4 Related Case

Related cases are concepts closely connected to, but distinct from, the main concept being studied; they share similarities but lack some defining attributes (Walker and Avant 2019). Examining related cases helps clarify the unique features of the main concept and its relationship with surrounding ideas (Walker and Avant 2019). Below is a fictional case designed by the authors. The related concepts were indicated in parentheses.

Mr. S.O., a 70‐year‐old patient, was admitted from the emergency department. Upon arrival at the ward, he was in stable condition but using diapers. The healthcare team encouraged ambulation (active movement; prevention of immobility‐related harm) since admission (early mobility) and performing basic activities of daily living, such as going to the bathroom, and removed his diaper (functional mobility). In this sense, the patient was instructed to mobilise progressively, monitoring his tolerance (progressive mobility), and to remain seated on the bed for a few minutes before standing up, in order to prevent episodes of orthostatic hypotension (safe gait). Mr. S.O., well oriented, always waited until after breakfast to go to the bathroom, avoiding episodes of hypoglycaemia and a potential fall with related injuries (safe mobility behaviour). A shower chair was provided (safe patient handling equipment). However, on the third day of hospitalisation, Mr. S.O. experienced constipation and, while straining to have a bowel movement, developed vasovagal hypotension. Since there were no grab bars in the bathroom, he fell forward and sustained a significant traumatic brain injury (TBI) (fall‐related harm).

There were included concepts that, although related, differ from ‘safe mobility’:

1. **Early mobility**: refers to initiating movement of the older adults as soon as clinically feasible, in order to maintain or improve functional capacity and prevent complications related to immobility (Braun et al. 2019; Lim et al. 2020);
2. **Functional mobility**: refers to moving independently, performing activities of daily living, which is crucial for maintaining independence and quality of life (Raymond et al. 2017);
3. **Progressive mobility**: refers to an approach to gradually increase the patient's activity level, starting from passive movements up to full ambulation, based on individual tolerance and clinical condition (Brown et al. 2016);
4. **Safe gaiety**: refers to the ability to walk in a way that minimises the risk of falls (Cheung et al. 2024);
5. **Safe mobility behaviour**: encompasses cognitive processes and protective actions used by individuals to prevent falls (Cheung et al. 2024);
6. **Safe patient handling equipment**: refers to equipment and devices designed to assist healthcare professionals in moving and repositioning patients safely, reducing the risk of injury both to patients and staff (Kumble et al. 2024).

#### 3.3.5 Invented Case

An invented case is a fictional scenario—often with elements of science fiction—used to illustrate a concept outside of its usual context (Walker and Avant 2019). It helps clarify the defining attributes of the concept, especially when the concept is very familiar or commonly taken for granted (Walker and Avant 2019). Below is a fictional case designed by the authors:

Mr. S.O., 70 years old, is admitted to a clinical ward, and upon admission, the nurse in charge—who is a specialist in gerontology—conducts an integrated clinical assessment with the support of artificial intelligence. The room where Mr. S.O. is accommodated is equipped with an interactive biofeedback mirror, which guides and corrects movements during exercises through therapeutic games that stimulate cognition and socialisation with other patients (active movement and prevent immobility‐related harm). The hospital uses an integrated smart flooring system that is cushioned, nonslip and pressure‐sensitive. It emits a soft auditory alert to the care team when it detects unstable steps (preventing fall‐related injuries).

#### 3.3.6 Illegitimate Case

An illegitimate case is one in which the concept term is used improperly or out of context, lacking most or all of the defining attributes (Walker and Avant 2019). It helps clarify the boundaries of the concept by illustrating what the concept is not (Walker and Avant 2019). Below is a fictional case designed by the authors:

The hospital where Mr. S.O. was admitted ensures mobility safety, as there are security cameras and 24‐h surveillance.

In the illegitimate case, the term ‘safe mobility’ was used in the sense of moving through the hospital without external threats, considering surveillance and security cameras. However, this does not apply to the concept under analysis, which is, actually, related to ensuring that older adults move actively while preventing harm associated with falls and immobility.

### 3.4 Antecedents and Consequences

Antecedents and consequences are essential attributes for refining the defining attributes (Walker and Avant 2019).

#### 3.4.1 Antecedents

Antecedents are events or incidents that must occur before the concept develops (Walker and Avant 2019). Table 2 presents the antecedents of the concept of ‘safe mobility’, with specific application in the hospitalised older adults, based on the literature reviewed.

**TABLE 2:** Antecedents of the concept of ‘safe mobility’, with specific application in hospitalised older adults, identified in the literature.

Antecedents	Studies in which the antecedents were identified
**1. Assessment and management of intrinsic conditions in older adults:** Cognitive status (dementia? delirium?), pathologies/signs/symptoms/comorbidities/conditions (stroke, anaemia, psychosis, congestive heart failure, vertigo, urinary incontinence, pain, dyspnoea, fatigue, weakness), visual acuity, gait quality and balance, nutritional status, functional capacity, sleep and muscle strength	Hendrich (2021); Krause and Von Renteln‐Kruse (2007); Golder et al. (2012); Boltz et al. (2014); Oliveira et al. (2016); Aarden et al. (2019); Resnick and Boltz (2019); Cerilo and Siegmund (2022); Labella et al. (2011); Aizen et al. (2015); Oliveira et al. (2016); Hartley et al. (2020); Seeger et al. (2021); Vivanti et al. (2011); Villafañe et al. (2015); Resnick and Boltz (2019); Courtney et al. (2011); Nogueira Lira et al. (2015); Brown et al. (2016); Braun et al. (2019); Mccullagh et al. (2020); Mahoney (1998); Lim et al. (2020); Gazineo et al. (2021); Mudge et al. (2022)
**2. Guarantee of a protective hospital environment:** (a) *through the use of technologies*: alarms, sensors, beds that allow height and side rail adjustments, hip protectors, wheelchairs suitable for weight/size and safety, belts/walking aids; (b) *through its infrastructure*: visual signage of the environment, clutter‐free environment, furniture arrangement, lighting, dry and nonslip flooring, grab bars in bathrooms, handrails on stairs; (c) *through its policies and management*: staff sizing compatible with the number and complexity level of older adults, legislation/values/norms/protocols, risk management programmes, provision of appropriate clothing	Zieschang et al. (2010); Nogueira Lira et al. (2015); Aizen et al. (2015); Oliveira et al. (2016); Lim et al. (2020); Hendrich (2021); Cerilo and Siegmund (2022); Mahoney (1998); Nelson et al. (2004); Hamers and Huizing (2005); Labella et al. (2011); Sinha and Detsky (2012); Boltz et al. (2014); Resnick and Boltz (2019); Krause and Von Renteln‐Kruse (2007); Brown et al. (2007); Seeger et al. (2021); Lyons (2014); Mudge et al. (2022); Wald et al. (2019); Hartley et al. (2020); Kneafsey et al. (2013); Mccullagh et al. (2020)
**3. Existence of a committed and trained multidisciplinary team:** who supervise patients, engage in decision‐making, possess technical‐scientific knowledge, are motivated, prescribe medications appropriately (avoiding polypharmacy and certain drug classes—antipsychotics, antidepressants, narcotics, benzodiazepines, diuretics), properly indicate the use and duration of invasive devices, promote early hospital discharge, assess and intervene in nutritional status, prescribe, conduct and supervise physical exercises	Mahoney (1998); Aizen et al. (2015); Mudge et al. (2022); Nelson et al. (2004); Brown et al. (2007); Labella et al. (2011); Boltz et al. (2014); Wald et al. (2019); Resnick and Boltz (2019); Seeger et al. (2021); Zieschang et al. (2010); Nogueira Lira et al. (2015); Lim et al. (2020); Mccullagh et al. (2020); Krause and Von Renteln‐Kruse (2007); Golder et al. (2012); Oliveira et al. (2016); Cerilo and Siegmund (2022); Hamers and Huizing (2005); Vivanti et al. (2011); Braun et al. (2019); Kneafsey et al. (2013); Lyons (2014)
**4. Older adult's behaviour:** Having a realistic perception of risks and their own capacity, adhering to the use of technologies, wearing appropriate footwear, having confidence and being correctly instructed	Aizen et al. (2015); Seeger et al. (2021); Cerilo and Siegmund (2022); Nelson et al. (2004); Brown et al. (2007); Boltz et al. (2014); Resnick and Boltz (2019); Krause and Von Renteln‐Kruse (2007); Mccullagh et al. (2020); Lyons (2014); Villafañe et al. (2015); Mahoney (1998); Oliveira et al. (2016); Lim et al. (2020)
**5. Family involvement:** Encouraging safe mobility practices, promoting a sense of well‐being and support	Nelson et al. (2004); Boltz et al. (2014); Lyons (2014); Resnick and Boltz (2019); Hendrich (2021); Mahoney (1998); Oliveira et al. (2016); Lim et al. (2020)

*Source:* The authors, 2025.

For the promotion of safe mobility among hospitalised older adults, certain prerequisites are necessary, that is, the antecedents of the concept. **Intrinsic conditions** (cognitive status, pathologies/signs/symptoms/comorbidities/conditions—stroke, anaemia, psychosis, congestive heart failure, vertigo, urinary incontinence, pain, dyspnoea, fatigue, weakness ‐, visual acuity, gait quality and balance, nutritional status, functional capacity, sleep and muscle strength) of older adults may compromise the active movement and increase the risk of fall‐related injuries (Lim et al. 2020). Therefore, intrinsic conditions should be identified and, if modifiable, addressed (Lim et al. 2020).

Additionally, a **safe environment** should be provided. The use of gerontechnology resources—such as alarms, sensors, beds with adjustable height and side rails, hip protectors, wheelchairs appropriate to the patient's weight/size and safety needs, as well as belts and walking aids—supports the promotion of older adults' autonomy, the maintenance of functional capacity and the prevention of harm related to immobility, providing safety during active movement (Hendrich 2021). Moreover, a safe hospital infrastructure—with visual signage, clutter‐free spaces, organised furniture, adequate lighting, dry and nonslip floors, grab bars in bathrooms and handrails on stairs—helps reduce the risk of fall‐related injuries. Institutional culture is another relevant aspect; its policies and management practices must take fall risk into account while also prioritising rehabilitation (Hendrich 2021). Institutions that place excessive emphasis on fall prevention—often establishing strict ‘zero falls’ goals—may inadvertently create a culture of fear and punishment among healthcare professionals (Hendrich 2021). This punitive focus can lead nurses to restrict patient mobility by keeping them in bed or discouraging ambulation, which contradicts best practices (Hendrich 2021). In addition, policies that do not reimburse costs associated with in‐hospital falls may further pressure healthcare teams to avoid any incidents, even if it means limiting patient mobility (Hendrich 2021). Such an approach can lead to adverse consequences, including functional decline, prolonged hospital stays and a higher risk of complications related to immobility (Hendrich 2021).

In this regard, a **multidisciplinary effort** is essential to combine skills and knowledge in collaboration with the patient to establish an intervention plan that promotes safe mobility and meets the individual needs of older adults (Kneafsey et al. 2013). Professionals who actively participate in clinical decision‐making, supported by solid technical‐scientific knowledge and a proactive approach to care; who prescribe exercise programmes as well as medications appropriately, avoiding polypharmacy and the unnecessary use of drug classes that increase the risk of fall‐related harm, such as antipsychotics, antidepressants, narcotics, benzodiazepines and diuretics; and who properly assess the indication and duration of invasive device use, which tends to confine patients to bed or impair their gait. Furthermore, the promotion of early hospital discharge by these professionals, when clinically appropriate, is also a strategy to reduce the risks of harm related to immobility (Kneafsey et al. 2013). Taken together, these actions contribute to a care approach that prioritises both safety and the quality of life of older adults.

Another important factor is **patient behaviour**: Most older adults fall when attempting to transfer, ambulate or go to the bathroom without assistance, often avoiding asking for help to avoid inconveniencing the nursing staff (Kneafsey et al. 2013). Additionally, many older adults believe they should remain in bed to ensure they are readily available to healthcare professionals, while others tend to restrict themselves to bed due to a fear of falling, particularly after experiencing a previous fall (Mahoney 1998). When not properly identified and addressed, this fear can trigger a cycle of immobility, increasing the risk of adverse events (Mahoney 1998).

Finally, **family involvement** and education are crucial; family members may, with good intentions, encourage a patient role that increases dependency and promotes bed rest without understanding the harmful effects of immobilisation (Mahoney 1998). On the other hand, by equipping families with the knowledge and skills to support mobility, the risk of functional decline and fall‐related injuries can be significantly reduced, both during hospitalisation and after discharge (Mahoney 1998). That is because the presence of family members can provide older adults with a sense of security, enhancing their confidence to move more independently and safely. This aligns with a patient‐centred approach that recognises the family as an integral partner in the care process (Mahoney 1998).

#### 3.4.2 Consequents

Consequents are events or incidents that occur as a result of the concept (Walker and Avant 2019). Table 3 presents the consequents of the concept of ‘safe mobility’, with specific application in the hospitalised older adults, based on the literature reviewed.

**TABLE 3:** Consequents of the concept of ‘safe mobility’, with specific application in the hospitalised older adults, identified in the literature.

Consequents	Studies in which the consequents were identified
**1. Maintenance of functionality:** Independence and autonomy	Seeger et al. (2021); Hendrich (2021); Gazineo et al. (2021); Resnick and Boltz (2019); Nogueira Lira et al. (2015); Lyons (2014); Nelson et al. (2004); Mudge et al. (2022); Brown et al. (2007); Sinha and Detsky (2012); Kneafsey et al. (2013); Boltz et al. (2014); Wald et al. (2019); Braun et al. (2019); Hartley et al. (2020); Lim et al. (2020); Hendrich (2021); Cerilo and Siegmund (2022); Mahoney (1998); Opasich et al. (2010); Zieschang et al. (2010); Vivanti et al. (2011); Labella et al. (2011); Courtney et al. (2011); Laybourne et al. (2011); Said et al. (2012); Golder et al. (2012); Villafañe et al. (2015); Brown et al. (2016); Raymond et al. (2017); Aarden et al. (2019); Lim et al. (2020); Mccullagh et al. (2020)
**2. Improved quality of life**: Increased safety and well‐being of the patient both inside and outside the hospital setting	Courtney et al. (2011); Kneafsey et al. (2013); Nelson et al. (2004); Boltz et al. (2014); Wald et al. (2019); Laybourne et al. (2011); Kneafsey et al. (2013); Lyons (2014); Villafañe et al. (2015); Resnick and Boltz (2019)
3. Reduction of hospital costs	Courtney et al. (2011); Sinha and Detsky (2012); Mahoney (1998); Zieschang et al. (2010); Kneafsey et al. (2013); Lyons (2014); Oliveira et al. (2016); Raymond et al. (2017)
4. Reduction of hospitalisation time	Sinha and Detsky (2012); Opasich et al. (2010); Vivanti et al. (2011); Labella et al. (2011); Courtney et al. (2011); Wald et al. (2019); Nelson et al. (2004); Mahoney (1998); Krause and Von Renteln‐Kruse (2007); Boltz et al. (2014); Lyons (2014); Oliveira et al. (2016); Resnick and Boltz (2019); Hartley et al. (2020); Lim et al. (2020); Mccullagh et al. (2020); Gazineo et al. (2021); Seeger et al. (2021); Mudge et al. (2022)
5. Reduction of unplanned hospital readmission rates	Courtney et al. (2011); Golder et al. (2012); Lyons (2014); Villafañe et al. (2015); Aarden et al. (2019); Braun et al. (2019); Resnick and Boltz (2019); Mccullagh et al. (2020); Gazineo et al. (2021); Hendrich (2021); Seeger et al. (2021)
6. Less need for long‐term care institutions (LTCI)	Mahoney (1998); Zieschang et al. (2010); Laybourne et al. (2011); Kneafsey et al. (2013); Boltz et al. (2014); Lyons (2014); Villafañe et al. (2015); Brown et al. (2016); Wald et al. (2019); Braun et al. (2019); Lim et al. (2020); Mccullagh et al. (2020); Gazineo et al. (2021); Cerilo and Siegmund (2022)
7. Reduction in mortality rates	Said et al. (2012); Laybourne et al. (2011); Kneafsey et al. (2013); Villafañe et al. (2015); Brown et al. (2016); Aarden et al. (2019); Braun et al. (2019); Hartley et al. (2020); Gazineo et al. (2021); Mudge et al. (2022)

*Source:* The authors, 2025.

**FIGURE 3:**
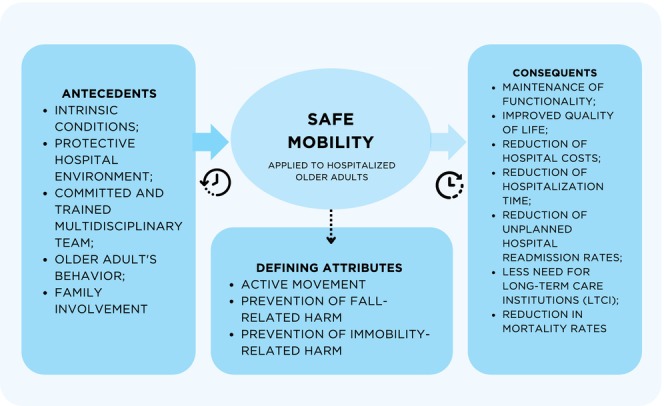
Connotations of the concept of ‘safe mobility’ as applied to hospitalised older adults. *Source:* Authors, 2025. [Colour figure can be viewed at wileyonlinelibrary.com]

By promoting safe mobility during hospitalisation, the functional decline accelerated by prolonged bed rest is prevented. In other words, the older adult preserves their autonomy and independence, which directly impacts their quality of life, both in the hospital setting and after discharge (Kneafsey et al. 2013). As safe mobility reduces the risk of harm related to falls and immobility, there are fewer complications and a lower risk of prolonged hospital stays, as well as reduced costs—optimising hospital resources and relieving pressure on health services (Kneafsey et al. 2013). When older patients maintain their functional capacity, they are more likely to continue aging in their own homes rather than being referred to long‐term care institutions (which often occurs when they become dependent and lack adequate family support) (Kneafsey et al. 2013). This is also reflected in lower rates of unplanned hospital readmissions and reduced mortality. Thus, safe mobility is not merely about fall prevention, but about promoting continuous and integrated rehabilitation that regards the older adult as an active subject in their care and a protagonist in their own recovery (Kneafsey et al. 2013) (Figure 3).

### 3.5 Empirical Referents

At the concluding stage of concept analysis, empirical referents corresponding to the defining attributes are identified (Walker and Avant 2019). These referents consist of categories or types of observable phenomena that, by their presence or occurrence, provide evidence that the concept is manifesting (Walker and Avant 2019). Rather than serving as tools to quantify the concept itself, empirical referents function as indicators through which the defining attributes can be recognised or evaluated. Therefore, they are directly associated with the attributes, not the concept (Walker and Avant 2019).

Safe mobility involves performing active movement through physical therapy sessions and exercises—strengthening, balance, stretching, aerobics, walking—as well as activities of daily living and socialisation. To assess these aspects, the following instruments are highlighted (Table 4):

**TABLE 4:** Empirical referents that enable the recognition of the defining attribute ‘active movement’.

Empirical referents	Description
Barthel Index	Measures the functional independence of an individual in 10 tasks—feeding, bathing, personal hygiene, dressing, bowel and bladder elimination, use of the toilet, transfers, ambulation and stairs (Krause and Von Renteln‐Kruse 2007; Zieschang et al. 2010; Lyons 2014; Brown et al. 2016; Hartley et al. 2020)
Katz Index	Assesses 6 functions: bathing, dressing, hygiene, transfers, continence, and feeding (Oliveira et al. 2016; Brown et al. 2016; Aarden et al. 2019)
Functional Independence Measure (FIM)	Assesses functional capacity in 18 activities divided into self‐care, mobility, locomotion, sphincter control, communication and cognition (Vivanti et al. 2011)
Activity Measure for Post‐Acute Care (AMPAC) 6‐Clicks	Assesses the need for assistance with bed mobility, sitting and standing from a chair, bed transfers, chair mobility, stair climbing, walking in the hospital room (Wald et al. 2019)
Banner Mobility Assessment Tool (BMAT)	Assesses the ability to transition from lying in bed to sitting, raise the arm at midline, raise the leg and extend the knee, flex the ankle and point toes, stand, march in place, step forward and backward; includes recommendations for safe patient handling based on observed mobility level and individual environment (Wald et al. 2019)
de Morton Mobility Index	Assesses mobility in bed, chair, static balance, walking and dynamic balance (Wald et al. 2019)
Hierarchical Assessment of Balance and Mobility	Assesses frail older adults for balance when sitting, standing, and walking; independence for transfers; maximum distance the patient can walk; assistance needed when walking (Wald et al. 2019)
Johns Hopkins Highest Level of Mobility	Assesses lying in bed, movement in bed, sitting, transfer to a chair, standing, walking ≥ 10 steps, walking ≥ 25 ft and walking > 250 ft (Wald et al. 2019)

*Source:* The authors, 2025.

Damage Reduction focuses on minimising severe injuries related to falls, such as fractures, sprains, lacerations and concussions resulting from falls. To map these risks, the following instruments are used (Table 5):

**TABLE 5:** Empirical references that enable recognition of the defining attribute ‘prevention of fall‐related harm’.

Empirical referents	Description
Functional Reach Test (FRT)	Assesses balance and postural stability, particularly in relation to the ability to maintain balance while reaching forward to grab an object (Vivanti et al. 2011)
Berg Balance Scale (BBS)	Assesses functional balance through 14 everyday tasks. Low scores indicate a higher risk of falls and consequently, injuries (Villafañe et al. 2015; Raymond et al. 2017)
Clinical Test of Sensory Interaction for Balance (CTSIB)	An assessment tool used to measure a person's ability to maintain balance under different sensory conditions (Golder et al. 2012)
Balance Performance Oriented Mobility Assessment (BPOMA)/Tinetti Test	A clinical tool used to assess balance and mobility in patients, particularly older adults at risk for falls (Opasich et al. 2010; Zieschang et al. 2010); Villafañe et al. 2015; Oliveira et al. 2016)
Gait Speed	An important measure of functional mobility and an individual's physical capacity, often used in clinical assessments to predict overall health, fall risk, and functional independence, especially in older adults (Raymond et al. 2017)
Time Up and Go test (TUG)	Measures the time it takes for the patient to stand from a chair, walk three meters, return and sit back down. Longer times suggest greater vulnerability to falls and injuries (Opasich et al. 2010; Vivanti et al. 2011; Laybourne et al. 2011; Said et al. 2012; Golder et al. 2012; Villafañe et al. 2015; Raymond et al. 2017; Braun et al. 2019; Hendrich 2021)
Chair Stand Test	A functional test used to assess lower limb strength, balance and functional capacity (Opasich et al. 2010)
Step Test Average	Used to assess motor function, balance and coordination ability through the capacity to ascend and descend steps (Vivanti et al. 2011)
Walking Impairment Questionnaire (WIQ)	An instrument that evaluates walking ability in individuals with conditions affecting mobility (Courtney et al. 2011)
Elderly Mobility Scale (EMS)	Assesses mobility through a series of items that measure the older person's ability to perform activities related to locomotion, balance and walking (Said et al. 2012)
Functional Ambulation Categories (FAC) score	A scale that categorises walking into six levels of ambulation (Braun et al. 2019)
Morton Mobility Index (DEMMI)	Assesses mobility and functional capacity of individuals, particularly those at risk of reduced mobility (Said et al. 2012; Aarden et al. 2019; Braun et al. 2019; Hartley et al. 2020)
Fall Risk Awareness Questionnaire (FRAQ)	A self‐assessment tool used to measure an individual's awareness of their fall risk (Cerilo and Siegmund 2022)
Assessment Instrument for Falls Among the Hospitalized Elderly (hospital AIFE)	Gathers individual aspects (identification; sensory organs; musculoskeletal system; activities of daily living assessment; balance and gait assessment; fall and fracture history) and environmental aspects (fall risk environmental scale) (Oliveira et al. 2016)
Fall Risk Environmental Scale	A scale composed of questions addressing the safety of movement areas, furniture arrangement, lighting, and accessibility to objects (Oliveira et al. 2016)
Hendrich II Fall Risk Model	A tool used to assess a patient's fall risk, particularly in healthcare settings (Hendrich 2021)

*Source:* The authors, 2025.

Damage reduction related to immobility can be mapped through items from the following instrument (Table 6):

**TABLE 6:** Empirical reference that enables recognition of the defining attribute ‘Prevention of immobility‐related harm’.

Empirical referents	Description
Frailty Index	A scale that assesses frailty based on the accumulation of deficits, such as restricted activity level, dependence on personal care and inability to frequently mobilise (Braun et al. 2019)

*Source:* The authors, 2025.

The use of these empirical references allows for a structured and evidence‐based approach to promoting safe mobility. The combination of these instruments enables not only the early identification of risk factors but also the implementation of personalised strategies to maintain the functionality and safety of hospitalised older adults.

### 3.6 Definition of the Concept

Based on the concept analysis, ‘safe mobility’, in the context of hospitalised older adults, refers to the patient's active movement carried out in a way that prevents the risk of harm related to falls and immobility. It is a multidimensional concept that involves elements related to the older adult (their conditions and behaviours), the institution (its infrastructure, culture and professionals), and the family. Safe mobility promotes functionality, enhances quality of life and improves health outcomes during and after hospitalisation, with positive results also observed at the institutional level.

## 4 Discussion

This concept analysis contributes to advancing theoretical clarity around the concept of ‘safe mobility’, with specific application to hospitalised older adults, and invites reflection on its implementation in real clinical settings. The prevailing institutional logic in hospitals often equates safety with immobility, driven by fear of liability, risk‐averse performance indicators, and the cultural consolidation of bed rest (Zisberg et al. 2023). Within this logic, older adults are viewed as ‘potential falls’ rather than as individuals with the right to move, recover and engage in their care (Zisberg et al. 2023). Thus, the concept of safe mobility, as revealed in this concept analysis, challenges the paradox at the core of geriatric hospital care: that attempts to protect older adults by immobilising them may, in fact, cause harm (Kok et al. 2021).

This analysis, therefore, understands safe mobility not as a clinical checklist, but as a complex sociotechnical practice—immersed in power relations, risk discourses and the (in)visibility of aging bodies in institutional settings (Growdon et al. 2017; Helvik 2021). In this sense, the concept has both epistemological and ethical implications. Epistemologically, it invites a shift from linear cause‐and‐effect thinking to a relational model of care, in which safe mobility is co‐constructed through interactions among patients, professionals, families, spaces and technologies (Hendrich 2021). Ethically, it requires the recognition of older adults not as passive recipients of protection, but as active agents whose autonomy must be respected—even when that entails calculated risk (Gao 2024).

The absence of a consolidated definition of safe mobility in nursing classifications such as NANDA‐I, NIC and NOC is not merely a taxonomic gap. It reflects a broader ontological invisibility of geriatric rehabilitation within the acute care settings (Yi et al. 2024). Without formal representation, mobility promotion remains vulnerable to being overshadowed by biomedical imperatives. Conceptual clarity, therefore, names safe mobility as a legitimate and necessary domain of nursing care and affirms its place in the hospital priority hierarchy.

Finally, future research involving critical implementation science is needed, along with interdisciplinary theorisation, to understand how aging bodies are managed in hospital spaces—thereby ensuring that safe mobility evolves from concept to practice.

## 5 Limitations

Although this concept analysis has provided valuable insights into ‘safe mobility’, with specific application to hospitalised older adults—a concept still relatively underexplored in nursing—it is not without limitations. A primary limitation is the scarcity of empirical literature directly addressing the concept, which constrained the depth and breadth of the analysis. Consequently, the defining attributes, antecedents, consequences and empirical referents identified in this study are not exhaustive. Another significant limitation is the lack of empirical validation of the concept in diverse clinical settings, which restricts the generalisability and practical applicability of the findings. Furthermore, cultural considerations were not fully explored, which may affect how ‘safe mobility’ is perceived and operationalised across different sociocultural contexts. These limitations highlight the need for further theoretical refinement and empirical studies to validate and expand the concept, ensuring it reflects the complex, multifaceted nature of safe mobility in older adults within varied healthcare environments.

## 6 Conclusion

The concept analysis revealed that safe mobility involves promoting active movement and preventing harm related to both immobility and falls. To achieve this, it requires a multidimensional approach that includes individualised clinical assessment, the involvement of trained professionals and family members, and an adequately structured hospital environment. Implementing this concept supports functional recovery, shortens hospital stays, reduces healthcare costs and lowers the risks of early institutionalisation, readmissions and mortality. Thus, safe mobility can be understood as a patient‐centred care practice focused on rehabilitation and the preservation of older adults' autonomy. The adoption of this concept fosters patient‐centred care, and future studies should explore its application in various clinical settings, recognising that concepts are dynamic and evolve over time.

## 7 Implications for Nursing Practice

Promoting safe mobility in nursing practice involves encouraging active movement and preventing harm related to falls and immobility while ensuring the maintenance of functional capacity in hospitalised older adults. This necessitates reviewing care protocols that restrict mobility and prioritising strategies that preserve patient functionality. Such practices help reduce immobility‐related complications, decrease hospital stays, prevent readmissions and optimise healthcare costs. Additionally, continuous professional education, multidisciplinary collaboration and patient and family engagement in the recovery process are essential to ensuring a care approach centred on patient functionality and quality of life.

## Author Contributions

Esther Mourão Nicoli: substantial contributions to the conception or design of the work; Drafting the work; Final approval of the version to be published; Agreement to be accountable for all aspects of the work in ensuring that questions related to the accuracy or integrity of any part of the work are appropriately investigated and resolved. Frances Valéria Costa e Silva: substantial contributions to the conception or design of the work; Reviewing the work critically for important intellectual content; Final approval of the version to be published; Agreement to be accountable for all aspects of the work in ensuring that questions related to the accuracy or integrity of any part of the work are appropriately investigated and resolved. Rosane Barreto Cardoso: acquisition, analysis, or interpretation of data for the work; Drafting the work; Final approval of the version to be published; Agreement to be accountable for all aspects of the work in ensuring that questions related to the accuracy or integrity of any part of the work are appropriately investigated and resolved. Tallita Mello Delphino: acquisition, analysis, or interpretation of data for the work; Reviewing the work critically for important intellectual content; Final approval of the version to be published; Agreement to be accountable for all aspects of the work in ensuring that questions related to the accuracy or integrity of any part of the work are appropriately investigated and resolved. Luciana Guimarães Assad: acquisition, analysis, or interpretation of data for the work; Reviewing the work critically for important intellectual content; Final approval of the version to be published; Agreement to be accountable for all aspects of the work in ensuring that questions related to the accuracy or integrity of any part of the work are appropriately investigated and resolved. Samara Gonçalves de Oliveira: acquisition, analysis, or interpretation of data for the work; Reviewing the work critically for important intellectual content; Final approval of the version to be published; Agreement to be accountable for all aspects of the work in ensuring that questions related to the accuracy or integrity of any part of the work are appropriately investigated and resolved.

## Conflicts of Interest

The authors declare no conflicts of interest.

## Supporting information


**Data S1.** Supporting Information.

## Data Availability

The data that supports the findings of this study are available in the Supporting Information of this article.
